# Prevalence of malarial recurrence and hematological alteration following the initial drug regimen: a retrospective study in Western Thailand

**DOI:** 10.1186/s12889-019-7624-1

**Published:** 2019-10-15

**Authors:** Manas Kotepui, Chuchard Punsawad, Kwuntida Uthaisar Kotepui, Voravuth Somsak, Nuoil Phiwklam, Bhukdee PhunPhuech

**Affiliations:** 1 Medical Technology Program, School of Allied Health Sciences, Walailak University, Nakhon Si Thammarat, 80160 Thailand; 2 School of Medicine, Walailak University, Nakhon Si Thammarat, 80160 Thailand; 3 Medical Technology Laboratory, Phop Phra Hospital, Phop Phra District, Tak Province 63160 Thailand

**Keywords:** Malaria, Malarial recurrence, Hematological parameters, Malarial drugs

## Abstract

**Background:**

The hematological changes following the initial drug regimen has been poorly understood in Thailand. This study was designed to determine the prevalence of malaria parasite recurrence and hematological alteration of patients during the initial drug regimen.

**Methods:**

A retrospective study was conducted at Phop Phra Hospital, Tak Province, located in northwestern Thailand. All data from patients who were diagnosed with *Plasmodium* spp. infection – including types of *Plasmodium* spp., clinical characteristics, and hematological parameters – were retrieved and analyzed.

**Results:**

The results demonstrated that during years 2012–2018, 95 out of 971 patients (9.78%) were infected with malaria two or more times. The gender, nationality, symptom of headache, type of *Plasmodium* spp., and career of each patient were associated with recurrence (*P*-value< 0.05). Among patients treated with malarial drug, the leukocyte count and red cell distribution width (RDW) were significantly changed when compared to untreated patients with recurrence (P-value< 0.05).

**Conclusion:**

This study indicated the high prevalence of malarial recurrence in Tak Province, Western Thailand, and its relationship to certain characteristics of individuals. Patients who were treated with antimalarial drugs exhibited leukocyte and RDW changes following the initial drug regimen. This data could be useful for prompt detection, treatment, and prevention of malarial recurrence in endemic areas of Thailand.

## Background

*Plasmodium* spp. causes human malaria, and it can cause persistent blood-stage infections lasting for weeks, months, and occasionally years [1]. *P. vivax* and *P. ovale* have a unique stage in the liver called hypnozoites, which remain arrested in the liver for weeks to years until they are activated to cause new blood-stage infections [2–4].

A recurrent infection is a newly detectable episode of blood-stage parasitemia occurring after a previous infection [3]. The recurrence in patients with malaria can be caused by reinfection from a new mosquito bite, recrudescence, or relapse [5]. Relapse occurs in *P. vivax* and *P. ovale* infections through the activation of hypnozoites in the human liver. Reinfections could be ignored in cases where patients move to an area with no malarial transmission [6]. Relapses could be ignored if patients are treated effectively with the full regimen of primaquine [7]. The source of recurrence could be explored by microscopic examination, genetic characterization of the parasites, and following quantification of drugs in the blood [8, 9].

Although the Bureau of Epidemiology, Ministry of Public Health is in charge of malaria control through the strategies of the Thailand National Malaria Elimination Plan in Thailand [10], there are people who still live in endemic areas of malaria with high infection rates for consecutive years, such as Tak Province [11]. These people might get malarial infection more than one time per season. In Africa, children suffered from malarial attacks every 4 to 6 weeks for many years, which has resulted in the spread of chloroquine resistance, the increase in the use of more toxic alternative drugs, and the exposure of other people living in the same endemic area to the side effects of antimalarial drugs [12, 13]. A previous report in Thailand provided evidence of the high proportion of recurrence in patients with *P. falciparum* and *P. vivax* infection at 21.5 and 31.5%, respectively [14]. Malarial recurrence also affects the neurological performance of children. Some studies showed short-term neurological impairment [15, 16] and long-term neurological impairment [17, 18] of patients with cerebral malaria after acute infection with the *P. falciparum* parasite. A previous study conducted among 457 Thai children indicated that low mean scores in mathematics and Thai language were in relation to higher numbers of malarial attacks [19]. Therefore, it is very important for the public health sector to implement strategies for early diagnosis, prompt treatment, vector control, and even behavioral changes to reduce incidences of malaria among the population living in endemic areas. Although countries in the Greater Mekong Subregion (GMS) played a role in regional malaria elimination which resulted in reducing the number of malarial cases, several malarial cases were still endemic among remote areas along international borders [11]. The hematological changes in malaria patients following the initial drug regimen are not well described. A previous study that performed routine checks on hematological parameters showed that cured patients had a higher mean hemoglobin level and lower neutrophil count at Day 28 compared to the baseline, where all had normal neutrophil counts on Day 0 [20]. Another study also indicated a decline in absolute neutrophil counts after amodiaquine, sulfadoxine/pyrimethamine, and amodiaquine-sulfadoxine/pyrimethamine combined were administered [21, 22].

Although previous studies have reported on the malarial incidences and hematological parameters in endemic areas of Thailand, they have not clarified on how the number of malarial recurrence might relate to the outcome of treatments and the transference of malaria to healthy individuals in the same area [11]. Moreover, the hematological changes following the initial drug regimen has been poorly understood in Thailand. In addition to the study of hematological changes, the study of the clinical characteristics of malaria patients was also performed to find any important factors related to malarial recurrence since previous studies indicated up to 80% of malaria-infected cases presented patients with headaches [23, 24].

This study was designed to determine the prevalence of malarial recurrence and hematological alteration during the initial drug regimen. The data might be useful for those organizations that play an important role in detection, treatment, and prevention of malaria parasites.

## Methods

Protocol for this study was approved by the Ethical Clearance Committee on Human Rights Related to Researches Involving Human Subjects of Walailak University, Thailand (EC number WUEC-18-145-01). A retrospective study was conducted at Phop Phra Hospital, Tak Province, located in northwestern Thailand. All data of patients diagnosed with *Plasmodium* spp*.* infection were retrieved from the laboratory information system (LIS) of the Medical Technology Laboratory, Phop Phra Hospital, Phop Phra District, Tak Province, Thailand. Criteria for eligibility included the following: single infection with *Plasmodium* spp. confirmed by microscopic examination. Laboratory records of the demographic characteristics, careers, signs and symptoms of each patient before admittance at the hospital, complete blood count parameters (CBC), and parasite density were also collected. Blood count parameters were performed using the BC-5200 Haematology Analyzer (Mindray, Nanshan, Shenzhen, China). The Analyzer provided data on white blood cells (WBCs), red blood cells (RBCs), hemoglobin (Hb) levels, platelet counts, mean corpuscular volume (MCV), mean corpuscular hemoglobin (MCH), mean corpuscular hemoglobin concentration (MCHC), RDW, and five part differentials [11].

The results of the thick blood smears, CBC, and signs and symptoms on the first day and on days following post-diagnosis were performed and collected routinely by the Medical Technology Laboratory during the time of each patient’s follow-up. All data were input in the Excel sheet and statistical software before trimming and analyzing. Recurrence of malaria was defined as a positive thick blood smear between Days 29 and 180, with or without clinical symptoms.

The process of data analysis began with defining the data types. Qualitative variables included demographic data, signs and symptoms, and types of *Plasmodium* spp. that were analyzed using the frequency, percentage, and Chi-squared test. Differences in hematological parameters in recurrent and non-recurrent malaria groups before and after the first drug regimen were analyzed by the Mann-Whitney U Test. Binary logistic regression was used to assess the prognosis value among risk factors associated with recurrence. All analyses were carried out using SPSS 11.5 for Windows, version 11.5, software (Chicago, SPSS Inc.).

## Results

The clinical and laboratory characteristics of patients are shown in Table 1. During years 2012–2018, 971 patients were infected with malaria. Among those patients, 95 patients (9.78%) were infected with malaria two or more times. The age range of patients who were infected with malaria was between one to ninety-one years old. The mean age for patients with recurrence and non-recurrence was 17.5 years and 20 years, respectively. Patients in this study included Thais (58.1%, 564/974) and non-Thais (41.9%, 410/974). The highest malarial cases were found in 2012 (31.5%, 306/971). Patients came through the Out Patients Department (OPD) (67%, 653/974) and the In Patients Department (IPD) (33%, 321/974). Patients were infected with *P. vivax* (74.5%, 726/974) and *P. falciparum* (25.5%, 248/974). They had a parasite density of few (50.4%, 491/974), moderate (34.6%, 337/974), and many (5.54%, 54/974). Most of them were students or self-employed in career (54.2%, 264/974).

**Table 1:** Clinical characteristics of patients

	Recurrence *n* = 95	Non-recurrence *n* = 876	*P*-value	OR (95%CI)
Demographic				
Age	17.5 (1–61)	20 (1–91)	0.124^a^	NA
Age groups (years)			0.199^b^	NA
< 5	4 (4.3%)	67 (7.7%)		
5–20	50 (53.2%)	373 (42.6%)		
21–40	26 (27.7%)	270 (30.9%)		
41–60	13 (13.8%)	128 (14.6%)		
> 60	1 (1.1%)	37 (4.2%)		
Male/Female, n (%)	70 (73.3%)/25 (26.3%)	539 (61.5%)/337 (38.5%)	0.02^b^	1.75 (1.09–2.82)
Thai/Non-Thai,n (%)	66 (69.5%)/29 (30.5%)	498 (56.8%), 378 (43.2%)	0.018^b^	1.727 (1.09–2.73)
House location			0.959^b^	NA
- Khirirat	2 (2.1%)	26 (3%)		
- Chong Khaep	9 (9.5%)	123 (14%)		
- Phop Phra	31 (32.6%)	304 (34.7%)		
- Mahawan	0	2 (0.2%)		
- Maeku	0	2 (0.2%)		
- Maejan	0	2 (0.2%)		
- Mogro	0	4 (0.5%)		
- Ruam Thai Phatthana	8 (8.4%)	75 (8.6%)		
- Wow Lay	45 (47.4%)	335 (38.2%)		
- Nong Ya Sai	0	1 (0.1%)		
- Na Bot	0	1 (0.1%)		
- Piang Luang	0	1 (0.1%)		
Year (cases, percent)			0.425	NA
2012 (306, 31.4%)	36 (11.8%)	270 (88.2%)		
2013 (204, 20.9%)	24 (11.8%)	180 (88.2%)		
2014 (132, 13.6%)	9 (6.9%)	123 (93.1%)		
2015 (120, 12.3%)	12 (10%)	108 (90%)		
2016 (81, 8.3%)	6 (7.4%)	75 (92.6%)		
2017 (59, 6.1%)	4 (7%)	53 (93%)		
2018 (71, 7.3%)	4 (5.6%)	67 (94.4%)		
Case admission	68 (10.4%)	585 (89.6%)	0.344^b^	1.25
- OPD	27 (8.5%)	291 (91.5%)		(0.78-
- Admit				1.99)
Days of fever (mean ± SD)	3.47 ± 1.92	3.83 ± 2.30	0.118^b^	NA
Chill (yes/no)	43 (45.7%)/51 (54.3%)	370 (42.2%)/506 (57.8%)	0.513^b^	1.15 (0.75–1.77)
Snot (yes/no)	21 (22.3%)/73 (77.7%)	173 (19.7%)/703 (80.3%)	0.551^b^	1.17 (0.7–1.95)
Headache (yes/no)	59 (62.8%)/35 (37.2%)	444 (50.7%)/432 (49.3%)	0.026^b^	1.64 (1.06–2.54)
Pain (yes/no)	17 (18.1%)/77 (81.9%)	190 (21.7%)/686 (78.3%)	0.418^b^	0.418 (0.8–1.38)
Type of *Plasmodium* spp. (Pf/Pv)	32 (33.7%)/63 (66.3%)	212 (24.2%)/663 (75.8%)	0.044^b^	1.59 (1.01–2.49)
Parasite density			0.915^b^	
- Rare	8 (9.2%)	79 (90.8%)		
- Few	48 (9.8%)	443 (90.2%)		
- Moderate	35 (36.8%)	302 (34.6%)		
- Many	4 (10.4%)	50 (89.6%)		
Career			0.001^b^	NA
- Student	41 (15.5%)	223 (84.5%)		
- Custody	7 (6.7%)	98 (93.3%)		
- Farmer	31 (10.1%)	275 (89.9%)		
- Self employed	15 (5.7%)	249 (94.3%)		
- Others	1 (3.1%)	31 (96.9%)		

^a^Comparison of continuous variable using Mann-Whitney U Test, ^b^Comparison of categorical groups using Pearson Chi-Square Test

According to the Chi-squared test that made an analysis between the clinical characteristics of patients and their recurrence status, the results demonstrated that the gender of patients was associated with recurrence (*P*-value = 0.020). Male patients had a higher risk of recurrence than female patients (Odd’s ratio = 1.75, 95% CI = 1.09–2.82). Nationality was also associated with recurrence (*P*-value = 0.018). Thai patients had a higher risk of recurrence than non-Thai patients (Odd’s ratio = 1.73, 95% CI = 1.09–2.73). The career of patients was associated with recurrence (*P*-value = 0.001). Prevalence of recurrence was frequently found in student groups (41/223, 15.5%) and farmer groups (31/275, 10.1%). The type of *Plasmodium* spp. was also associated with recurrence (*P*-value = 0.044). Patients with *P. falciparum* infection had a greater risk of recurrence (Odd’s ratio = 1.588, 95% CI = 1.01–2.49). Patients who came with headaches had a greater risk of recurrence than those with non-headaches (P-value = 0.026, Odd’s ratio = 1.64, 95% CI = 1.58–2.54). In this study, the age group of patients was not associated with recurrence (P-value > 0.05). The highest recurrence cases were at 6–20 (53.2%) and 21–40 (27.7%) years old, respectively. The recurrences were found mostly in Wow Lay (47.4%, 45/95), Phop Phra Subdistrict (32.6%, 31/95), Chong Khaep (9.5%, 9/95), Ruam Thai Phatthana (8.4%, 8/95), and Khirirat (2.1%, 2/95).

Hematological alterations following the initial drug regimen have been investigated, and the results are shown in Table 2. The results showed that neutrophil, lymphocyte, and eosinophil percentages were significantly changed (*P*-value< 0.05). The mean neutrophil percentage (54.9%) after treatment with the initial drug regimen was lower than before treatment (70.1%). The mean lymphocyte percentage (32.7%) following the initial drug regimen was higher than before treatment (20.2%). RBC parameters, RBC count, Hb, hematocrit, MCV, and MCH were significantly lower following the initial drug regimen compared to before treatment (*P*-value< 0.05). Platelet counts were significantly higher following the initial drug regimen compared to before treatment (P-value = 0.007).

**Table 2:** Differences in hematological parameters in recurrent and non-recurrent malaria groups before and after first drug regimen

Parameter	Malarial recurrence (Mean ± SD)		*P*-value	Non-recurrence		*P*-value
	*First admission*	*First follow up*		*First admission*	*First follow up*	
Leukocyte(× 10^3^/μL)	6.19 (2.02)	6.36 (2.16)	0.81	6.21 (2.41)	6.36 (2.16)	0.014
Neutrophil (×10^3^/μL)	70.4 (12.9)	54 (12.4)	< 0.001*	70.4 (14.44)	54 (12.4)	< 0.001
Lymphocyte (×10^3^/μL)	19.8 (10.9)	34.4 (12.2)	< 0.001*	20.1 (12.2)	34.4 (12.2)	< 0.001
Monocyte (× 10^3^/μL)	6.03 (4.13)	7.11 (3.68)	0.182	6.09 (6.23)	7.11 (3.68)	< 0.001
Eosinophil (×10^3^/μL)	2.69 (2.42)	3.04 (2.08)	0.093	2.32 (1.91)	3.24 (3.28)	< 0.001
Basophil (×10^3^/μL)	1.05 (0.86)	1.38 (1.09)	0.357	1.00 (0.86)	1.38 (1.09)	< 0.001
RBC (×10^6^/μL)	4.44 (0.92)	4.3 (0.7)	0.001*	4.59 (0.74)	4.3 (0.7)	< 0.001
Hemoglobin (g/dL)	12.1 (2.52)	11.7 (1.96)	< 0.001*	12.4 (2.67)	11.7 (1.97)	< 0.001
MCV (fL)	81.9 (6.41)	80.2 (9.07)	< 0.001*	80.1 (8.12)	80.2 (9.07)	0.017
MCH (pg/cell)	29.4 (9.7)	27.4 (3.29)	0.015*	27.2 (3.14)	27.4 (3.29)	0.006
MCHC (g/dL)	34.2 (0.79)	33.8 (2.4)	0.155	33.9 (1.16)	33.8 (2.4)	0.708
RDW (%)	12.5 (0.73)	13 (4.38)	0.603	12.6 (1.21)	13 (4.38)	< 0.001
Platelet (×10^3^/μL)	84.75 (38.2)	168.5 (133.7)	0.007*	91.5 (51.1)	168.5 (133.7)	< 0.001

*P-value by Mann-Whitney U Test

### Prediction of malarial recurrence

For the prediction of reinfection, multinomial logistic regression had been performed, and the results are shown in Table 3. This table provided the regression coefficient (B), the Wald statistic (to test the statistical significance), and the all-important Odds Ratio (Exp (B)) for each variable category. The gender, nationality, types of *Plasmodium* spp., symptom of headache, and career of each patient were investigated in the regression equation. The results showed that there was a significant overall effect (*P*-value = 0.027). The overall Wald statistic for all parameters was 4.88. The B coefficients for all parameters except nationality were significant and negative, indicating that decreasing affluence was associated with increased odds of achieving malarial recurrence.

**Table 3:** Regression analysis of factors related to malarial recurrence

Analysis type	Variable	Malarial recurrence			
		B^a^	Wald	*P*-value	Exp (B)
Multivariate	Gender	−0.543	4.88	0.027	0.581
	Nationality	−0.412	4.69	0.088	0.663
	Career	−0.334	2.92	< 0.0001	0.716
	Type of *Plasmodium* spp.	−0.556	12.67	0.021	0.574
	Headache	−0.548	5.33	0.017	0.578
	Constant	1.618	5.73	0.027	–

^a^unstandardized variables

The Exp (B) column (the Odds Ratio) indicated that female patients were 0.581 times more likely than male patients to achieve malarial recurrence. Comparatively, no significance of effect was found in nationality (*P*-value = 0.088). The effect of career was also significant and negative, indicating that farmers, nonages, employees, and other careers were less likely to achieve malarial recurrence than student groups. The OR indicated that they were 0.716 times less likely to achieve malarial recurrence. In comparing between two types of *Plasmodium* spp., a significance of effect was found (*P*-value = 0.021). The effect of the type of *Plasmodium* spp. was also significant and negative, indicating that those infected with *Plasmodium falciparum* were less likely to achieve malarial recurrence than those infected with *Plasmodium vivax*. The OR indicated that they were 0.574 times less likely to achieve malarial recurrence. In comparing between patients with headaches and non- headaches, a significance of effect was found (*P*-value = 0.017). The effect of headaches was also significant and negative, indicating that patients with non-headaches were less likely to achieve malarial recurrence than patients with headaches. The OR indicated that they were 0.578 times less likely to achieve malarial recurrence.

The key results demonstrated that all parameters tested including gender, type of *Plasmodium* spp., symptom of headache, and career can be candidates for prediction of malarial recurrence in a regression model (P-value < 0.05). The regression model (probability of recurrence) was 1.618 + (− 0.543)(gender) + (− 0.556) (type of *Plasmodium* spp.) + (− 0.548)(headache) + (− 0.334)(career).

## Discussion

This study investigated the prevalence, etiology, and hematological alteration of malaria patients with recurrence during the past 6 years from 2012 to 2017. Among the 6 years investigated, the highest frequency of recurrence was found during 2012–2013. However, the prevalence of recurrence continuously decreased from 10 to 5.6% during 2015–2018. This indicated very well the surveillance and control of malaria through the strategies of the Thailand National Malaria Elimination Plan [10]. A previous study reported that 24.1% of patients with *P. vivax* infection had at least one recurrence within 180 days of full treatment, and this was caused by therapeutic failure [25]. Other studies reported that the incidence of recurrence was 0 to 13.5% after treatment with standard malarial regimen [26–28].

This study found that male patients had a higher risk of malarial recurrence than female patients. The previous study reported that men have a greater risk of contracting malaria than women because of working in places where mosquitoes are numerous, such as in mines, fields, or forests at peak biting times [29]. Sleeping habitats of men, for example sleeping outdoors, may increase their risk of exposure to mosquitoes as well [30]. Some studies indicated that women lacked economic support from family to access health care for children with malaria [31, 32]. In regard to the correlation between the age of patients and the risk of recurrence in this study, more than half of the recurrence cases were in teenagers or school-aged children (6–20 years old). This result was correlated with the previous studies in western Kenya [33, 34]. Moreover, our study indicated that the recurrences were frequently found in Wow Lay, Phop Phra Subdistrict, Chong Khaep, Ruam Thai Phatthana, and Khirirat. It was very urgent to evaluate the effectiveness of community programs to prevent malarial recurrence and to use existing knowledge on practices to control the malaria burden in these endemic areas of malaria in Thailand.

This study demonstrated that Thai patients had a higher risk of recurrence than non-Thai patients. Most of the recurrence were in patients of Thai nationality (69.5%) compared to non-Thai nationality (30.5%). The major cause of recurrence among Thai nationals might be due to locally acquired infection among the community. In addition, Tak Province, located in the Thailand-Myanmar border, has been a malarial hotspot in Thailand for ten consecutive years. The recurrence among non-Thai nationals might be due to laborers and refugees moving across freely and annually around these areas. Previous studies indicated that this movement might make this area one of the most significant transmission areas of malaria in Thailand [35–37]. A previous study in this area also found that 50% of malaria patients were Thais, while 29% were migrants from Myanmar [38].

This study demonstrated that patients with the symptom of headaches had a higher risk of recurrence than patients with no sign of headaches. Some studies noted that up to 80% of malaria-infected cases presented patients with headaches [23, 24]. The mechanism of headaches in malaria is not well described. The cytokine is believed to be an important factor that might lead to headaches during malarial infection, especially acute malarial infection. One study demonstrated that malarial pathogenesis was associated with excessive production of pro-inflammatory cytokines, such as the tumor necrosis factor which induces headaches [39].

For hematological differences between patients with and without recurrence, patients with non-recurrence had a higher leukocyte count following the initial drug regimen, but no changes were observed in patients with recurrence. This study also showed significantly higher monocyte and basophil percentages with lower eosinophil percentages in patients with non-recurrence when compared to patients with recurrence. This suggests the role of monocytes, basophils, and eosinophils in the recurrence process. The previous study conducted in northwestern Thailand showed that monocyte, eosinophil, and basophil counts were significantly lower in patients with malaria when compared to those without malaria [11]. However, some studies indicated a higher monocyte count in children infected with malaria [40, 41]. A previous study reported that malarial infection could lead to low eosinophil levels in the early stage of the infection, and then increase over the following few weeks [42]. Moreover, a robust eosinophilic response after completion of antimalarial therapy is a predictor of a good recovery from malaria-associated anemia and suggests an important interrelationship between the immune response and erythropoiesis in the setting of malaria [43]. The previous study also indicated that the total leukocyte, lymphocyte, and neutrophil counts were improved following standard treatment [44]. This study indicated that lymphocyte percentages of both patients with recurrence and those with non-recurrence were significantly higher following the initial drug regimen compared to before treatment. This was correlated with the results of a previous study indicating that lymphocytosis reverted from lymphopenia in a matter of days after the initiation of antimalarial therapy [45]. However, a previous study indicated that T cell lymphopenia can be frequently found and played a key role in the pathogenesis of malaria, especially in *P. falciparum* infections [44]. Another hematological parameter, alteration of RDW, was observed in patients with non-recurrence following the initial drug regimen, but no changes were observed in patients with recurrence. The previous study showed that RDW in combination with other hematological parameters could increase the probability of malarial infection [46]. Another study indicated that patients with *P. falciparum* malaria, *P. vivax* malaria, and non-malaria infected groups had no significant differences in the median of RDW [11]. The alteration of RDW in this study may indicate that patients with recurrence might have a higher rate of erythropoiesis than patients with non-recurrence.

## Conclusion

This study indicated the high prevalence of malarial recurrence in Tak Province, Western Thailand, and its relationship to certain characteristics of individuals. Patients who were treated with antimalarial drugs exhibited leukocyte and RDW changes following the initial drug regimen. This data could be useful for prompt detection, treatment, and prevention of malarial recurrence in endemic areas of Thailand.

## Data Availability

The datasets used during the current study are available from the corresponding author based on reasonable request.

## Abbreviations

CBC: Complete Blood Count Parameters
CI: Confidence Interval
GMS: Greater Mekong Subregion
Hb: Hemoglobin
IPD: In Patients Department
LIS: Laboratory Information System
MCH: Mean Corpuscular Hemoglobin
MCHC: Mean Corpuscular Hemoglobin Concentration
MCV: Mean Corpuscular Volume
OPD: Out Patients Department
OR: Odd’s Ration
RBCs: Red Blood Cells
RDW: Red cell distribution width
WBCs: White Blood Cells

## References

[1] Ashley EA, White NJ (2014). The duration of Plasmodium falciparum infections. Malar J.

[2] White NJ (2011). Determinants of relapse periodicity in Plasmodium vivax malaria. Malar J.

[3] Battle KE, Karhunen MS, Bhatt S, Gething PW, Howes RE, Golding N (2014). Geographical variation in Plasmodium vivax relapse. Malar J.

[4] Lover AA, Coker RJ (2013). Quantifying effect of geographic location on epidemiology of Plasmodium vivax malaria. Emerg Infect Dis.

[5] White NJ, Imwong M (2012). Relapse. Adv Parasitol.

[6] Chen N, Auliff A, Rieckmann K, Gatton M, Cheng Q (2007). Relapses of Plasmodium vivax infection result from clonal hypnozoites activated at predetermined intervals. J Infect Dis.

[7] Robinson LJ, Wampfler R, Betuela I, Karl S, White MT, Li Wai Suen CS (2015). Strategies for understanding and reducing the *Plasmodium vivax* and Plasmodium ovale hypnozoite reservoir in Papua New Guinean children: a randomised placebo-controlled trial and mathematical model. PLoS Med.

[8] WHO. Methods for surveillance of antimalarial drug efficacy. Geneva: World Health Organization; 2009. Available from: http://apps.who.int/iris/bitstream/10665/44048/1/9789241597531_eng.pdf. Date cite 13 Feb 2019.

[9] Escalante AA, Ferreira MU, Vinetz JM, Volkman SK, Cui L, Gamboa D (2015). Malaria molecular epidemiology: lessons from the international centers of excellence for malaria research network. Am J Trop Med Hyg.

[10] President's malaria initiative, Thailand, Lao PDR, and Regional, Malaria Operational Plan FY 2018 2018. Available from: https://www.pmi.gov/docs/default-source/default-document-library/malaria-operational-plans/fy-2018/fy-2018-thailand-regional-malaria-operational-plan.pdf?sfvrsn=8. Date cite 15 Feb 2019.

[11] Kotepui M, Phunphuech B, Phiwklam N, Chupeerach C, Duangmano S (2014). Effect of malarial infection on haematological parameters in population near Thailand-Myanmar border. Malar J.

[12] Rogier C, Imbert P, Tall A, Sokhna C, Spiegel A, Trape JF (2003). Epidemiological and clinical aspects of Blackwater fever among African children suffering frequent malaria attacks. Trans R Soc Trop Med Hyg.

[13] Rono J, Farnert A, Murungi L, Ojal J, Kamuyu G, Guleid F (2015). Multiple clinical episodes of Plasmodium falciparum malaria in a low transmission intensity setting: exposure versus immunity. BMC Med.

[14] Douglas NM, Nosten F, Ashley EA, Phaiphun L, van Vugt M, Singhasivanon P (2011). Plasmodium vivax recurrence following falciparum and mixed species malaria: risk factors and effect of antimalarial kinetics. Clin Infect Dis.

[15] Meremikwu MM, Asindi AA, Ezedinachi E (1997). The pattern of neurological sequelae of childhood cerebral malaria among survivors in Calabar, Nigeria. Cent Afr J Med.

[16] Boivin MJ (2002). Effects of early cerebral malaria on cognitive ability in Senegalese children. J Dev Behav Pediatr.

[17] Holding PA, Stevenson J, Peshu N, Marsh K (1999). Cognitive sequelae of severe malaria with impaired consciousness. Trans R Soc Trop Med Hyg.

[18] Carter JA, Mung'ala-Odera V, Neville BG, Murira G, Mturi N, Musumba C (2005). Persistent neurocognitive impairments associated with severe falciparum malaria in Kenyan children. J Neurol Neurosurg Psychiatry.

[19] Vorasan N, Pan-Ngum W, Jittamala P, Maneeboonyang W, Rukmanee P, Lawpoolsri S (2015). Long-term impact of childhood malaria infection on school performance among school children in a malaria endemic area along the Thai-Myanmar border. Malar J.

[20] Adjuik M, Agnamey P, Babiker A, Borrmann S, Brasseur P, Cisse M (2002). Amodiaquine-artesunate versus amodiaquine for uncomplicated Plasmodium falciparum malaria in African children: a randomised, multicentre trial. Lancet..

[21] Staedke SG, Kamya MR, Dorsey G, Gasasira A, Ndeezi G, Charlebois ED (2001). Amodiaquine, sulfadoxine/pyrimethamine, and combination therapy for treatment of uncomplicated falciparum malaria in Kampala, Uganda: a randomised trial. Lancet..

[22] Olliaro P, Nevill C, LeBras J, Ringwald P, Mussano P, Garner P (1996). Systematic review of amodiaquine treatment in uncomplicated malaria. Lancet..

[23] Suyaphun A, Wiwanitkit V, Suwansaksri J, Nithiuthai S, Sritar S, Suksirisampant W (2002). Malaria among hilltribe communities in northern Thailand: a review of clinical manifestations. Southeast Asian J Trop Med Public Health.

[24] Faiz MA, Rahman MR, Hossain MA, Rashid HA (1998). Cerebral malaria--a study of 104 cases. Bangladesh Med Res Counc Bull.

[25] Zuluaga-Idarraga L, Blair S, Akinyi Okoth S, Udhayakumar V, Marcet PL, Escalante AA (2016). Prospective study of Plasmodium vivax malaria recurrence after radical treatment with a Chloroquine-Primaquine standard regimen in Turbo. Colombia Antimicrob Agents Chemother.

[26] Abdon NP, Pinto AY, das Silva Rdo S, de Souza JM (2001). Assessment of the response to reduced treatment schemes for vivax malaria. Rev Soc Bras Med Trop.

[27] Bergonzoli G, Rivers Cuadra JC (2000). Therapeutic efficacy of different antimalarial regimens in the Costa Rica-Nicaragua border region. Rev Panam Salud Publica.

[28] Rajgor DD, Gogtay NJ, Kadam VS, Kocharekar MM, Parulekar MS, Dalvi SS (2014). Antirelapse efficacy of various Primaquine regimens for Plasmodium vivax. Malar Res Treat.

[29] Reuben R (1993). Women and malaria--special risks and appropriate control strategy. Soc Sci Med.

[30] A/Rahman SH, Mohamedani AA, Mirgani EM, Ibrahim AM (1996). Gender aspects and women's participation in the control and management of malaria in Central Sudan. Soc Sci Med.

[31] Tolhurst R, Nyonator FK (2006). Looking within the household: gender roles and responses to malaria in Ghana. Trans R Soc Trop Med Hyg.

[32] Muller I, Smith T, Mellor S, Rare L, Genton B (1998). The effect of distance from home on attendance at a small rural health Centre in Papua New Guinea. Int J Epidemiol.

[33] Kepha S, Nikolay B, Nuwaha F, Mwandawiro CS, Nankabirwa J, Ndibazza J (2016). Plasmodium falciparum parasitaemia and clinical malaria among school children living in a high transmission setting in western Kenya. Malar J.

[34] Sultana M, Sheikh N, Mahumud RA, Jahir T, Islam Z, Sarker AR (2017). Prevalence and associated determinants of malaria parasites among Kenyan children. Trop Med Health.

[35] Kar NP, Kumar A, Singh OP, Carlton JM, Nanda N (2014). A review of malaria transmission dynamics in forest ecosystems. Parasit Vectors.

[36] Carrara VI, Lwin KM, Phyo AP, Ashley E, Wiladphaingern J, Sriprawat K (2013). Malaria burden and artemisinin resistance in the mobile and migrant population on the Thai-Myanmar border, 1999-2011: an observational study. PLoS Med.

[37] Bhumiratana A, Intarapuk A, Sorosjinda-Nunthawarasilp P, Maneekan P, Koyadun S (2013). Border malaria associated with multidrug resistance on Thailand-Myanmar and Thailand-Cambodia borders: transmission dynamic, vulnerability, and surveillance. Biomed Res Int.

[38] Epidemiology Bo. Annual epidemiological surveillance report 2011.

[39] Clark IA, Jacobson LS (1998). Do babesiosis and malaria share a common disease process?. Ann Trop Med Parasitol.

[40] Maina RN, Walsh D, Gaddy C, Hongo G, Waitumbi J, Otieno L, et al. Impact of *Plasmodium falciparum* infection on haematological parameters in children living in Western Kenya. Malar J. 2010, 9(Suppl 3):S4. 10.1186/1475-2875-9-S3-S4 PMC3002140 21144084

[41] Abdalla SH (1988). Peripheral blood and bone marrow leucocytes in Gambian children with malaria: numerical changes and evaluation of phagocytosis. Ann Trop Paediatr.

[42] Tangpukdee N, Yew HS, Krudsood S, Punyapradit N, Somwong W, Looareesuwan S (2008). Dynamic changes in white blood cell counts in uncomplicated Plasmodium falciparum and P. vivax malaria. Parasitol Int.

[43] Camacho LH, Wilairatana P, Weiss G, Mercader MA, Brittenham GM, Looareesuwan S (1999). The eosinophilic response and haematological recovery after treatment for Plasmodium falciparum malaria. Tropical Med Int Health.

[44] Rodriguez-Morales AJ, Sanchez E, Arria M, Vargas M, Piccolo C, Colina R (2005). White blood cell counts in Plasmodium vivax malaria. J Infect Dis.

[45] Hviid L, Kemp K (2000). What is the cause of lymphopenia in malaria?. Infect Immun.

[46] Jairajpuri ZS, Rana S, Hassan MJ, Nabi F, Jetley S (2014). An analysis of hematological parameters as a diagnostic test for malaria in patients with acute febrile illness: an institutional experience. Oman Med J.

